# Relationship between *Helicobacter pylori* Infection and Plasmacytoid and Myeloid Dendritic Cells in Peripheral Blood and Gastric Mucosa of Children

**DOI:** 10.1155/2019/7190596

**Published:** 2019-11-11

**Authors:** Anna Helmin-Basa, Małgorzata Wiese-Szadkowska, Anna Szaflarska-Popławska, Maciej Kłosowski, Milena Januszewska, Magdalena Bodnar, Andrzej Marszałek, Lidia Gackowska, Jacek Michalkiewicz

**Affiliations:** ^1^Department of Immunology, Collegium Medicum Nicolaus Copernicus University, Bydgoszcz 85-094, Poland; ^2^Department of Pediatric Endoscopy and Gastrointestinal Function Testing, Collegium Medicum Nicolaus Copernicus University, Bydgoszcz 85-094, Poland; ^3^Department of Clinical Pathomorphology, Collegium Medicum Nicolaus Copernicus University, Bydgoszcz 85-094, Poland; ^4^Department of Otolaryngology and Laryngological Oncology, Poznan University of Medical Science, Poznan 61-866, Poland; ^5^Chair of Oncologic Pathology and Prophylaxis, Poznan University of Medical Sciences & Greater Poland Cancer Center, Poznan 61-866, Poland; ^6^Department of Microbiology and Immunology, The Children's Memorial Health Institute, Warsaw 04-730, Poland

## Abstract

**Purpose:**

To investigate the frequency and activation status of peripheral plasmacytoid DCs (pDCs) and myeloid DCs (mDCs) as well as gastric mucosa DC subset distribution in *Helicobacter pylori*- (*H. pylori*-) infected and noninfected children.

**Materials and Methods:**

Thirty-six children were studied; twenty-one had *H. pylori*. The frequencies of circulating pDCs (lineage^−^HLA-DR^+^CD123^+^) and mDCs (lineage^−^HLA-DR^+^CD11c^+^) and their activation status (CD83, CD86, and HLA-DR expression) were assessed by flow cytometry. Additionally, the densities of CD11c^+^, CD123^+^, CD83^+^, CD86^+^, and LAMP3^+^ cells in the gastric mucosa were determined by immunohistochemistry.

**Results:**

The frequency of circulating CD83^+^ mDCs was higher in *H. pylori*-infected children than in the noninfected controls. The pDCs demonstrated upregulated HLA-DR surface expression, but no change in CD86 expression. Additionally, the densities of gastric lamina propria CD11c^+^ cells and epithelial pDCs were increased. There was a significant association between frequency of circulating CD83^+^ mDCs and gastric lamina propria mDC infiltration.

**Conclusion:**

This study shows that although *H. pylori*-infected children had an increased population of mature mDCs bearing CD83 in the peripheral blood, they lack mature CD83^+^ mDCs in the gastric mucosa, which may promote tolerance to local antigens rather than immunity. In addition, this may reduce excessive inflammatory activity as reported for children compared to adults.

## 1. Introduction

*Helicobacter pylori* (*H. pylori*) colonizes the human stomach. Infection usually starts in early childhood and can persist for decades, sometimes even for the lifetime [1]. *H. pylori*-infected children develop gastric mucosal inflammation [2] but with much lower infiltration of polymorphonuclear and mononuclear cells and decreased incidence of gastric and duodenal ulceration, gastric adenocarcinoma, and gastric mucosa-associated lymphoid tissue (MALT) lymphoma as compared to adults [3, 4].

Dendritic cells (DCs) are professional antigen-presenting cells (APCs) that induce T cell response against a presented antigen. Their migration from the site of an antigen uptake to the regional lymph nodes and their subsequent maturation profile decide about the type of the subsequent adaptive immune response profiles against infection (protective or nonprotective responses). The DC maturation process is associated with the expression of several membrane molecules engaged in the DC migration (CCR7), antigen presentation abilities (MHC class II), and their costimulatory activities (CD80, CD83, and CD86) [5, 6].

Human DCs are rare in peripheral blood [7] and consist of two major subsets, myeloid DCs (mDCs) and plasmacytoid DCs (pDCs) [8]. The mDCs express myeloid antigen such as CD11c and can be subdivided into CD1c- and CD141-bearing cells. pDCs lack these markers and instead express CD123, CD303, and CD304 molecules. Both mDC and pDC subsets display APC capacity, but this function is reduced in pDCs, which have relatively low expression of HLA-DR [9]. It is well documented that pDCs promote the generation of regulatory T cells (Tregs) [10].

The circulating DC number and their activation status may change during the course of infection [11, 12] and may alter throughout a lifetime [13]. These cells play an important role in shaping the anti-infection response by constantly completing the population of tissue-residing DCs.

The interaction of DCs with *H. pylori in vitro* induces DC maturation and release of proinflammatory cytokines, such as IL-6, IL-8, IL-12, and IL-23 [14–21]. However, the DC cytokine production profile and the T cell response mode strongly depend on the *H. pylori* structure [22]. The DCs expressing CD11c^+^ [18, 23, 24], the semi-mature DCs bearing dendritic cell-lysosome-associated membrane glycoprotein (DC-LAMP, or LAMP3), and the DCs with high HLA-DR but little CD80, CD83, and CD86 expression [25, 26] were found in proximity to and in lymphoid follicles, together with FoxP3 T cells, in the *H. pylori*-infected gastric mucosa of adults [26].

In contrast to many studies concerning DC participation in *H. pylori*-induced immune response in adults [23, 24, 26–30], similar ones in children are still rare [31]. Additionally, estimation of the frequencies of mDCs and pDCs and the expression levels of CD83, CD86, and HLA-DR in the peripheral DC subsets have not yet been evaluated in the *H. pylori*-infected children. There is also a paucity of information regarding the expression profile of the human DC maturation markers (LAMP3 and CD83), the myeloid cell marker (CD11c), and the pDC marker (CD123) in the gastric mucosa of *H. pylori*-infected children. Therefore, the aim of this study was (a) to investigate the distribution and activation status of peripheral and gastric mucosa DC subsets (pDCs and mDCs) in the *H. pylori*-infected and noninfected children and (b) to evaluate correlations between the DC subset phenotype characteristics and the activity score on the basis of the updated Sydney gastritis classification system.

## 2. Materials and Methods

### 2.1. Ethics Statement

The study was undertaken according to Helsinki declaration with ethics committee approval of Collegium Medicum Nicolaus Copernicus University in Bydgoszcz, Poland, and informed consent was obtained from all the patients' parents and from the patients older than 16 years prior to blood and antral biopsy collection.

### 2.2. Patients

In total, thirty-six subjects with dyspeptic symptoms, older than eleven years of age, were included in this study. The exclusion criteria involved the following: (1) a history of antibiotic use during 4 weeks for other reasons than *H. pylori* infection; (2) the presence of other inflammatory diseases, such as celiac disease, inflammatory bowel disease, or allergy; and (3) gastric perforation or hemorrhage, history of surgery, bleeding conditions, or evidence of other clinical conditions or intestinal parasites. Each subject or subject's parents provided the clinical history.

All the children were examined with gastroduodenal endoscopy due to permanent abdominal pain. Based on previous observation that *H. pylori*-associated gastritis of the corpus was present only when antral gastritis was present [32], three antral biopsies were taken from each patient. One biopsy specimen was subjected to a rapid urease test. The second specimen was used for histological analysis. The last specimen was subjected to immunohistochemical staining.

The urease test was performed for every patient. Furthermore, *H. pylori* infection was excluded in every subject by use of the ^13^C urea breath test, performed within one week of undergoing endoscopy. ^13^C concentration was measured with an infrared radiation analyzer (OLYMPUS Fanci2, Tokyo, Japan), assuming 4‰ as the cutoff point.

A patient was considered *H. pylori*-infected when the ^13^C urea breath test and either the rapid urease test or microscopic evaluation were positive for *H. pylori*. A patient was considered noninfected when all three tests were negative. Twenty-one patients were found to be *H. pylori* positive, while fifteen patients satisfied the criteria for a negative *H. pylori* status. None of the patients had ulcer disease or macroscopic lesions of the duodenal mucosa upon endoscopic examination.

The study was conducted in the *H. pylori*-infected and noninfected children and adolescents. The group of noninfected children (controls) consisted of 7 boys and 8 girls (median age 15 years, range 11-18 years). The group of *H. pylori*-infected children comprised 9 boys and 12 girls with recognized gastritis and *H. pylori* infection (median age 15 years, range 12-18 years). None of the patients had a history of other inflammatory diseases or cancer.

### 2.3. Histological Assessment

The specimen was formalin-fixed and embedded in paraffin, sectioned and stained with hematoxylin and eosin for histological analysis, or Giemsa modified by Gray stain for *H. pylori* detection. Biopsy specimens were graded for gastritis by two independent pathologists based on the updated Sydney system.

Histological variables (presence and density of mononuclear and polymorphonuclear cells, glandular mucosa atrophy, intestinal metaplasia, and *H. pylori*) were scored on a four-point scale: 0, none; 1, mild; 2, moderate; and 3, marked.

### 2.4. Flow Cytometry Analyses

During endoscopy, 5 mL of venous blood was taken for immunological testing. The frequencies of total DCs (tDCs), mDCs and pDCs, as well as the expression of CD83, CD86, and HLA-DR were determined in whole blood samples with the use of a five-color flow cytometry method. We followed the methods of Helmin-Basa et al. [33]. First, two tubes containing 200 *μ*L whole blood were stained with FITC Lin-1 lineage cocktail (antibodies against human CD3, CD14, CD16, CD19, CD20, and CD56), PerCP-anti-human HLA-DR, APC-anti-human CD83, PE-Cy7-anti-human CD86, PE-anti-human CD11c, or PE-anti-human CD123 (BD Biosciences, Franklin Laker, New Jersey, USA) antibodies for 30 min at room temperature (RT). The erythrocytes were removed with the use of BD Bioscience FACS Lysing Solution (BD Biosciences). The stained leukocytes were suspended in phosphate-buffered saline (PBS) and analyzed immediately with flow cytometry (FACSCanto II, BD Biosciences). The instrument was set up using a BD Cytometer Setup and Tracking beads. Data acquisition was performed using BD FACSDiva software, version 6.1.3 (BD Biosciences) and the analysis with FlowJo 7.5.5 (Tree Star Inc., Ashland, Oregon, USA). Compensation settings were performed with a set of compensation tubes using the BD CompBead antibody-capturing particles and the compensation setup tool with BD FACSDiva software.

**Figure 1:**
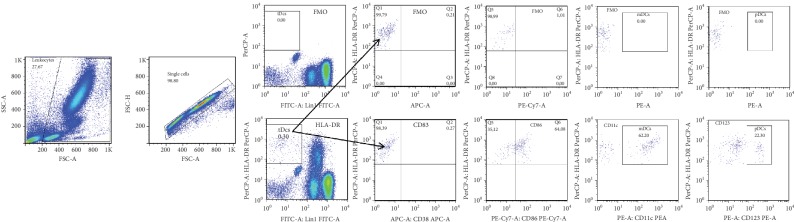
Fluorescence minus one controls for the quantification of dendritic cell subsets. Fluorescence minus one (FMO) controls were prepared without adding a particular fluorochrome-conjugated antibody, for instance fluorescence minus fluorescein (FITC), fluorescence minus allophycocyanin (APC), fluorescence minus tandem conjugate of phycoerytrin (PE), and the *cyanine* dye Cy7 (PE-Cy7), and fluorescence minus PE, respectively. The gating for each channel was defined to exclude nearly all backgrounds from the FMO controls on the 2D plots of HLA-DR versus individual fluorescence.

**Figure 2:**
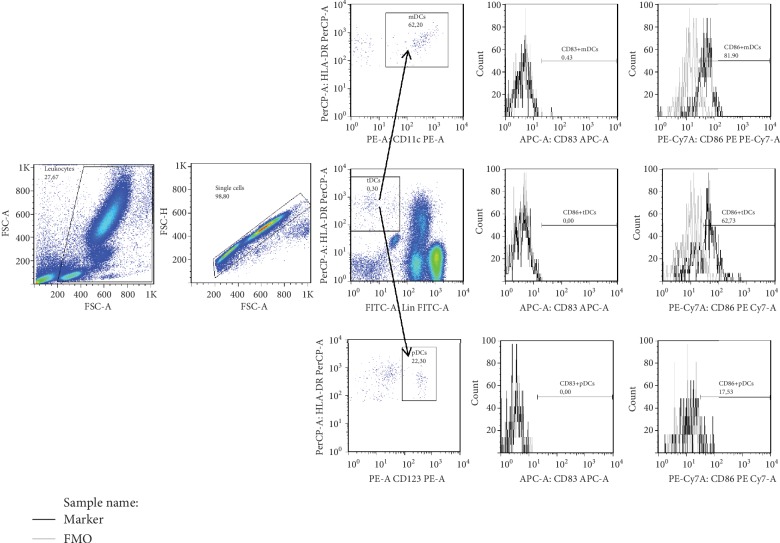
Gating strategy for multicolor flow cytometric detection of total dendritic cells and their subsets. At first, leukocytes were gated by forward scatter characteristic (FSC) versus side scatter characteristics (SSC). Next, doublets and aggregates were avoided by selecting singlet cells on a forward scatter: FSC (area)/FSC (height) plot. Then total dendritic cells (tDCs) were gated for fluorescence intensity of HLA-DR peridinin-chlorophyll proteins (PerCP) versus lineage-1 fluorescein (FITC), and using the fluorescence intensity of CD11c phycoerytrin (PE) or CD123 PE, myeloid dendritic cells (mDCs) or plasmacytoid dendritic cells (pDCs) were gated (open histrograms). Then in tDCs and their subsets, a percentage of CD83^+^ and CD86^+^ cells was gated (open histograms.) The grey line represents the staining profile for fluorescence minus one (FMO) control.

Fluorescence minus one (FMO) was used for gating (Figure 1). The gating strategy applied for the DC subset enumeration in two separate tubes is shown in Figure 2. Doublets and aggregates were avoided by selecting singlet cells on a forward scatter: FSC (area)/FSC (height) plot. Logical gating was used to identify tDC, mDC, and pDC populations. tDCs were defined as Lin1^−^HLA-DR^+^, mDCs were defined as Lin-1^−^HLA-DR^+^CD11c^+^ cells, while pDCs were defined as Lin-1^−^HLA-DR^+^CD123^+^ cells. The geometric mean fluorescence intensity (gMFI) was determined to quantify cell surface expression of activation molecules. Statistical analyses were based on at least 5000 events gated on the mDCs and 2500 events gated on the pDCs.

### 2.5. Immunohistochemistry

The immunohistochemical studies were performed on archived formalin-fixed paraffin-embedded (FFPE) tissue sections delivered to the Department of Clinical Pathomorphology Collegium Medicum in Bydgoszcz, Nicolaus Copernicus University in Torun.

FFPE tissue sections were cut on a manual rotary microtome (AccuCut, Sakura, Torrance, USA). Paraffin sections (4 *μ*m) were prepared and mounted onto extra adhesive slides (SuperFrost Plus, Menzel Glasser, Braunschweig, Germany).

The immunohistochemical staining was performed according to previously described procedures [34, 35] and standardized using a series of positive and negative control reactions. The positive control reaction was performed on a model tissue selected according to reference sources (The Human Protein Atlas, http://www.proteinatlas.org) [36] and the antibody datasheets. The negative control reactions were performed on additional tissue sections, by substituting the primary antibody with a solution of 1% bovine serum albumin (BSA) diluted in PBS.

Deparaffinization, rehydration, and antigen retrieval were performed by heating sections in Epitope Retrieval Solution high-pH at 95-98°C for 20 min (Dako, Agilent Technologies, Santa Clara, USA) in PT-Link (Dako, Agilent Technologies). Subsequently, endogenous peroxidase activity was blocked with the use of a 3% H_2_O_2_ solution for 15 min at RT and the nonspecific binding was blocked using 5% solution of BSA for 15 min at RT. Incubation with the primary antibodies, anti-LAMP3 (1 : 400 dilution, rabbit polyclonal), anti-CD11c (1 : 1000 dilution, rabbit monoclonal), anti-CD123 (IL-3RA, 1 : 200 dilution, rabbit polyclonal), anti-CD83 (1 : 100 dilution, mouse monoclonal), and anti-CD86 (1 : 50 dilution, rabbit monoclonal) (all from Abcam, Cambridge, England), at 4°C for 16 hours was performed. The antibody complex was detected using EnVision Flex Anti-Mouse/Rabbit HRP-Labeled Polymer (Dako, Agilent Technologies) and localized using 3-3′diaminobenzidine (DAB) as the chromogen. Finally, tissue sections were counterstained in hematoxylin, subsequently dehydrated, and cleared in a series of xylene washes, and a cover slip was applied using mounting medium (Dako, Agilent Technologies).

#### 2.5.1. Evaluation of Protein Expression Based on Immunohistochemical Staining

The evaluation of protein expression in the antrum area of obtained FFPE tissue sections was performed at 20x and 40x original objective magnifications, using the ECLIPSE E400 (Nikon Instruments Europe, Amsterdam, Netherlands) light microscope.

For the evaluation of expression, immunohistochemical reactions were scored according to morphometric principles based on a Remmele–Stegner scale (IRS-Index Remmele–Stegner; immunoreactive score) [37], used in our previous publications [34, 38].

The total immunoreactivity score was defined according to the scale obtained from the grade of intensity multiplied by the score of positively stained cells to give a total score from 0 to 12. The intensity of staining was scored as follows: 0, negative; 1, low staining; 2, moderate staining; and 3, strong staining. The number of positive cells was categorized as follows: (1) in the gastric lamina propria: 0—negative, 1—1 to 5 positive cells, 2—6 to 10 positive cells, 3—11 to 50 positive cells, and 4—51 to 100 positive cells, and (2) in epithelium mucosa: 0—negative, 1—1 to 20 positive cells, 2—21 to 50 positive cells, 3—51 to 80 positive cells, and 4—81 to 100 positive cells.

### 2.6. Statistical Analyses

All data are expressed as medians with the first and third quartiles. The results were compared using Mann-Whitney *U* test calculated by the STATISTICA 12 software (StatSoft). For normal distribution, variables were analyzed using the Kolmogorov–Smirnov test with Lilliefors correction. The age and gastric inflammation scores were correlated with the frequency of DC and their subsets using the Spearman's rank correlation. Statistical significance was considered at *p* < 0.05.

## 3. Results

### 3.1. Gastric Mucosa Histology

The intensity and activity of antral gastritis were greater in the *H. pylori*-infected children (*p* < 0.001 and *p* = 0.003, respectively) when compared to the *H. pylori* noninfected ones (Table 1). Interestingly, three children with *H. pylori* infection had mild gastric mucosal atrophy in the antrum. However, no intestinal metaplasia was seen in the children's gastric mucosa.

### 3.2. The Distribution of Circulating tDCs, mDCs, and pDCs and Their Activation Status in *H. pylori*-Infected and Noninfected Children

**Figure 3:**
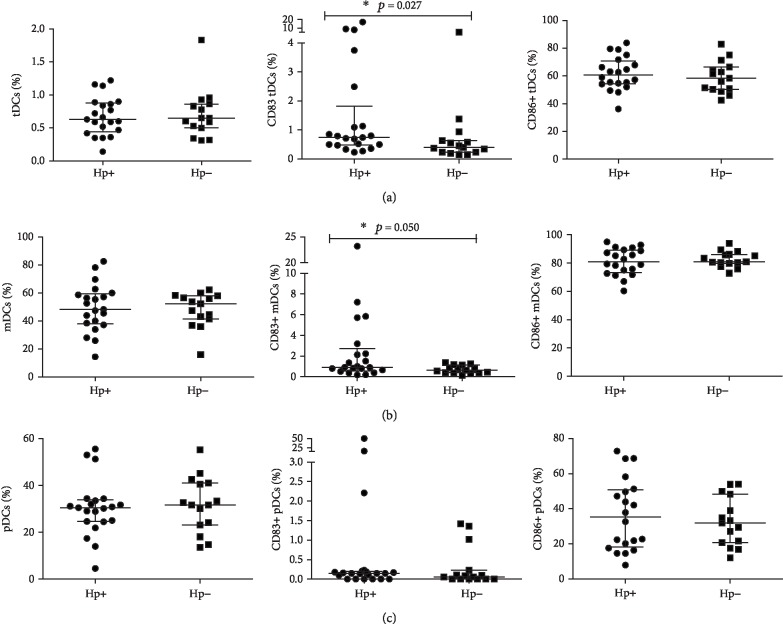
Comparison of the distribution of circulating total myeloid and plasmacytoid dendritic cells in *H. pylori*-infected and noninfected children with using individual raw data points. Scatter plots are showing differential percentages of total dendritic cells (tDCs), CD83^+^ tDCs and CD86^+^ tDCs (a), myeloid dendritic cells (mDCs), CD83^+^ mDCs and CD86^+^ mDCs (b), and plasmacytoid dendritic cells (pDCs), CD83^+^ pDCs and CD86^+^ pDCs (c), in *H. pylori*-infected children (Hp+: *n* = 21) compared to noninfected children (Hp-: *n* = 15). Data shown as individual raw data points, medians (horizontal line), and interquartile ranges (upper and lower whiskers). ^∗^Statistically significant differences (Mann-Whitney *U* test).

The percentages of tDCs, mDCs, and pDCs were similar between the two groups (Figures 3(a)–3(c)). While the percentages of CD83 expressing tDCs and mDCs were markedly higher in *H. pylori*-infected children (0.74% versus 0.40%, *p* = 0.027 and 0.92% versus 0.62%, *p* = 0.050), there was no significant difference in the percentage of CD83-positive pDCs between these two groups. The percentages of CD86 expressing tDCs, mDCs and pDCs were similar between *H. pylori*-infected and noninfected children (Figures 3(a)–3(c)) (*p* > 0.05).

**Figure 4:**
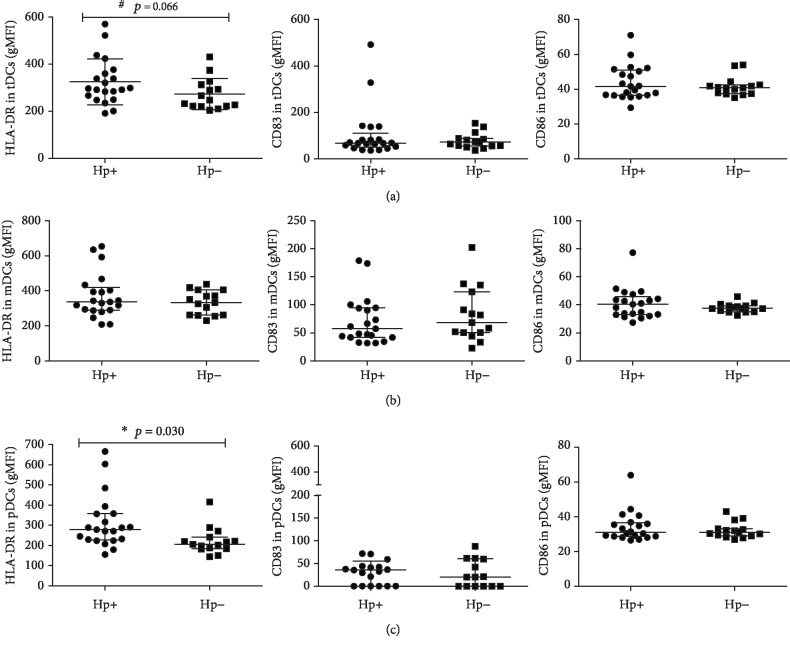
Comparison of activation status of total myeloid and plasmacytoid dendritic cells in *H. pylori*-infected and noninfected children with using individual raw data points. Scatter plots are showing differential densities (gMFI (geometric mean fluorescence)) of HLA-DR, CD83, and CD86 markers on the surface of total dendritic cells (tDCs) (a), myeloid dendritic cells (mDCs) (b) and plasmacytoid dendritic cells (pDCs) (c) in *H. pylori*-infected children (Hp+: *n* = 21) compared to noninfected children (Hp-: *n* = 15). Data shown as individual raw data points, medians (horizontal line), and interquartile ranges (upper and lower whiskers). ^∗^Statistically significant differences (Mann-Whitney *U* test).

Increased densities of HLA-DR on the surface of tDCs and pDCs were noted in *H. pylori*-infected children compared with noninfected controls (296.10 versus 247.51, *p* = 0.066 and 278.28 versus 204.89, *p* = 0.030, respectively) (Figures 4(a) and 4(c)). There were no statistically significant differences in CD83 and CD86 expression between the two groups (*p* > 0.05) (Figures 4(a)–4(c)).

### 3.3. The Densities of CD11c^+^, CD123^+^, CD83^+^, CD86^+^, and LAMP3^+^ Cells in the Gastric Mucosa of *H. pylori*-Infected and Noninfected Children

To determine the effect of *H. pylori* infection on the local immune system, the gastric mucosa sections were labeled with the following antibodies: anti-CD11c (high expression on mDCs, but low on granulocytes, macrophages, and a subset of B cells), CD123 (a marker of pDCs), CD83 and LAMP3 (markers of mature DCs), and CD86 (costimulatory molecule upregulated on activated DCs and other APCs).

**Figure 5:**
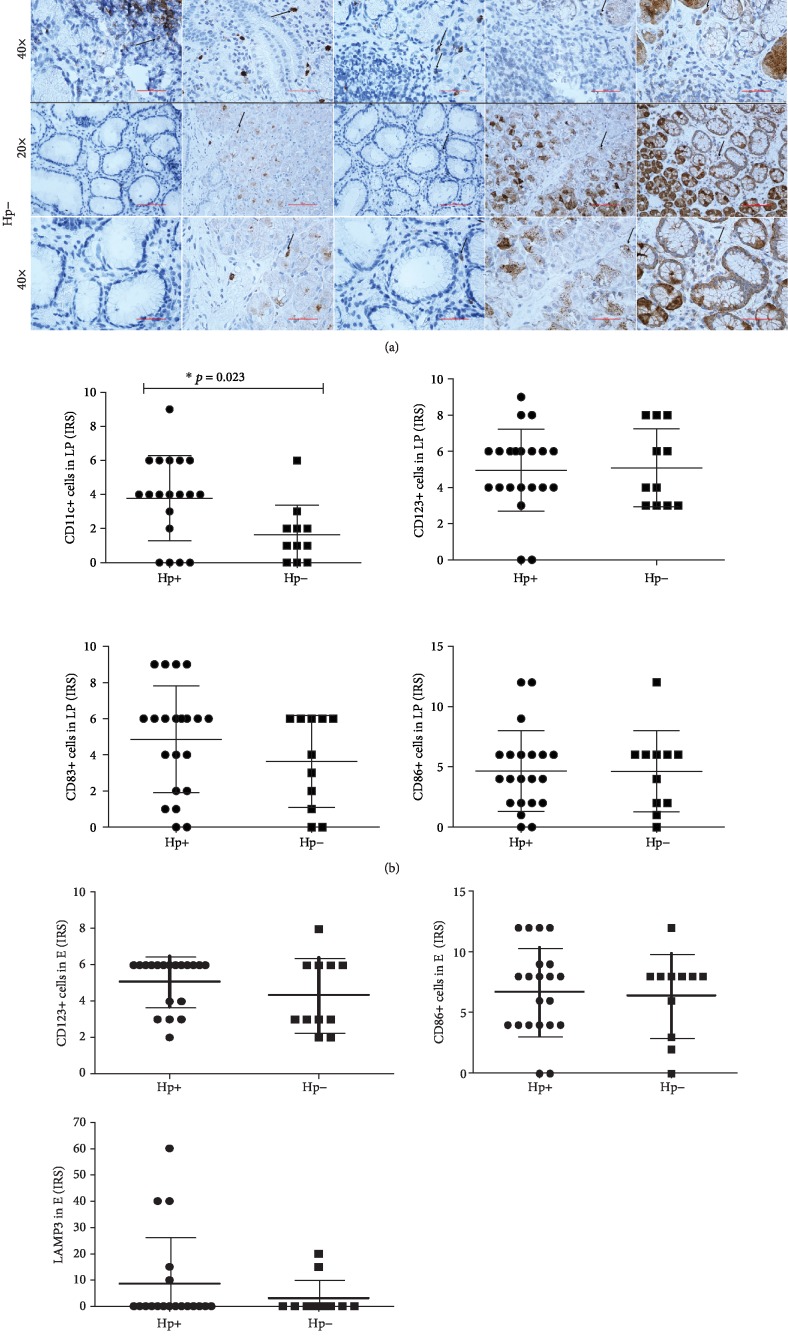
Infiltration of dendritic cells into the gastric mucosa in *H. pylori*-infected and noninfected children. Representative images of CD11c, CD123, CD83, LAMP3, and CD86 staining in the lamina propria and epithelium in antral gastric mucosa biopsies from *H. pylori*-infected (Hp+) and noninfected (Hp-) children. Specific staining appears in brown, and the cell nuclei are counterstained with hematoxylin in blue. The arrows indicate positive staining. Visualization was performed by immunohistochemistry. Primary objectives, 20x and 40x (a). Scatter plots are showing differential densities (IRS (immunoreactive score)) of CD11c^+^, CD123^+^, CD83^+^, and CD86^+^ cells in gastric lamina propria mucosa (b) and CD123^+^, CD86^+^, and LAMP3^+^ cells in gastric epithelium (c) in *H. pylori*-infected children (Hp+: *n* = 21) compared to noninfected children (Hp-: *n* = 11). Data shown as individual raw data points, medians (horizontal line), and interquartile ranges (upper and lower whiskers). ^∗^Statistically significant differences (Mann-Whitney *U* test).

A statistically significant increase in the accumulation of CD11c-positive cells was noted in the gastric lamina propria mucosa of *H. pylori*-infected children (*p* = 0.023) (Figures 5(a) and 5(b)). There was no significant difference between CD83^+^, CD86^+^, and CD123^+^ cells in *H. pylori*-infected and noninfected children (*p* > 0.05). However, the data display a tendency toward an increased density of CD123^+^ DCs in the gastric epithelium mucosa of *H. pylori*-infected children (Figures 5(a)– 5(c)) (*p* > 0.05). Moreover, we used immunohistochemistry to detect LAMP3^+^ cells in an antrum. We observed these cells but only in mucosal epithelium of six children with *H. pylori* infection and 2 noninfected children (Figures 5(a) and 5(c)).

### 3.4. Correlation Analysis

To evaluate whether *H. pylori*-associated gastritis influences DC frequency or whether these values depend more on age, we correlated age, gastric inflammation, and *H. pylori* density scores with the circulating and gastric DC frequencies.

**Figure 6:**
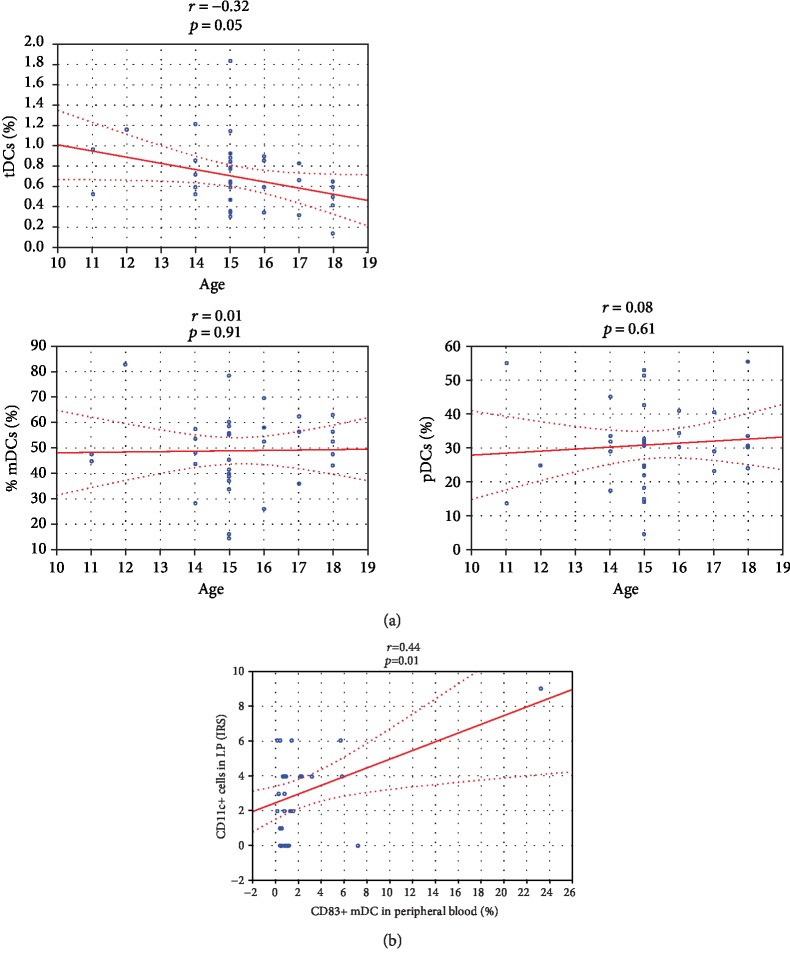
The relationship between the age and frequencies of dendritic cells and their subsets in *H. pylori*-infected and noninfected children. The relationships between the age and the frequencies of circulating total dendritic cells (tDCs), myeloid dendritic cells (mDCs), and plasmacytoid dendritic cells (pDCs) in *H. pylori*-infected and noninfected children were correlated by Spearman's correlation test (*p* < 0.05) (a). The relationship between the frequency of CD83^+^ myeloid dendritic cells (CD83^+^ mDCs) in peripheral blood and the density of CD11c^+^ dendritic cells (CD11c^+^ cells) in lamina propria (LP) in *H. pylori*-infected and noninfected children was correlated by Spearman's correlation test (*p* < 0.05) (b). IRS: immunoreactive score.

There was a weak inverse association between age and tDC frequency (*r* = ‐0.32, *p* = 0.05), but no effect of age on frequencies of mDC (*r* = 0.01, *p* > 0.05) and pDC (*r* = 0.08, *p* > 0.05) subsets was observed (Figure 6(a)). However, we did not observe any correlation between age and density of gastric DCs (*p* > 0.05).

There was no significant association between frequencies of circulating tDCs, their subsets (mDCs and pDCs), gastritis intensity (mononuclear cell infiltrate), its activity (polymorphonuclear cell infiltrate), and *H. pylori* density (*p* > 0.05). Additionally, no correlation was noted between gastric DCs frequencies, gastric inflammation scores, and *H. pylori* density (*p* > 0.05).

There was only a moderate positive correlation between frequency of circulating CD83^+^ mDCs and density of gastric lamina propria CD11c^+^ cells (*r* = 0.44 and *p* = 0.01, respectively) (Figure 6(b)).

## 4. Discussion

We described here peripheral DC subset distribution (mDCs and pDCs) and their maturation status (expression of CD83, CD86, and HLA-DR) in the *H. pylori*-infected and noninfected children. Additionally, the densities of CD11c^+^ (mDCs), CD123^+^ (pDCs), CD83^+^ or LAMP3^+^ (mature DCs), and CD86^+^ (activated) cells in the gastric mucosa were determined by immunohistochemistry. Similar to the other studies on *H. pylori* infection, our control group consisted of dyspeptic, noninfected patients [39, 40], because gastroduoendoscopy is not considered an ethical procedure in the noncomplaining subjects.

*H. pylori*-infected children exhibited (1) an increased percentage of circulating CD83^+^ mDCs, but with normal expression of CD86 and HLA-DR, (2) upregulation of HLA-DR expression but unchanged expression of CD83 and CD86 on pDCs subset, (3) increased densities of CD11c^+^ cells in the lamina propria and CD123^+^ cells in the epithelium, and (4) a significant association between frequency of circulating CD83^+^ mDCs and lamina propria infiltrating CD11c^+^ DCs.

The gastric mucosa, which does not initially have organized and diffuse lymphoid tissue, acquires MALT-like structures with follicles and germinal centers following contact with *H. pylori* [41]. *H. pylori* infection in children is associated with a higher accumulation of lymphoid follicles containing CD4^+^ T cells, B cells, and DCs [42] in the lamina propria of the gastric mucosa, identified by macroscopically evident nodularity.

*H. pylori* bacteria attach to the gastric epithelium and contact the gastric epithelium and subepithelial lamina propria DCs (direct or indirect), which rapidly increase in the *H. pylori-*infected mucosa compared with an uninfected mucosa [18, 23, 24]. DCs loaded with *H. pylori* likely migrate to gastric lymphoid follicles or draining paragastric lymph nodes to present *H. pylori* antigens to naive CD4^+^ T cells [43, 44], thus initiating and regulating the host *H. pylori-*specific immune response.

Three subsets of circulating DCs exist: the CD123^+^ pDCs and the two types of CD11c^+^ mDCs:CD1c^+^ mDCs with CD11c^low^ and CD141^+^ mDCs with CD11c^high^ [8]. Since DCs are rare in human peripheral blood [7], we focused on (a) the enumeration of the two major DC subsets (CD11c^+^ mDCs and CD123^+^ pDCs) and (b) the estimation of the correlation between these DC phenotypes, *H. pylori*-induced inflammation and *H. pylori* density scores, according to the updated Sydney system of gastritis classification. Additionally, we used immunohistochemistry to assess DC subset distribution in antral biopsy specimens.

The distribution of circulating DC subsets in *H. pylori*-infected patients has not yet been previously evaluated, and to our knowledge, this is the first study to demonstrate an increased percentage of CD83^+^ mDCs, but with no significant differences in surface expression of activation markers (CD86 and HLA-DR). Terminally differentiated DCs should induce CD83-specific maturation markers and increase the expression of CD86 costimulatory molecule and HLA-DR. Thus, an increase in the frequency of CD83-expressing mDCs along with the absence of upregulation of CD86 and HLA-DR molecules on mDCs might suggest that the maturation status of circulating mDCs in *H. pylori*-infected children was changed but only within the small part of mDCs. However, the rest of circulating mDCs still expressed immature/tolerogenic phenotype (HLA-DR^low^CD86^low^).

The peripheral blood is not the best source for the studying of DC maturation status in the course of *H. pylori* infection; hence, we studied also the gastric epithelial and lamina propria DC subset distribution and its maturation markers.

We observed high levels of CD11c-positive cells and a tendency toward increased density of CD83-positive cells in the gastric lamina propria of *H. pylori*-infected children. CD11c is a marker for DCs of myeloid origin (mDCs) which are potent APCs and can activate T cells. CD83 is a more selective marker, present on mature DCs. Hence, we used CD11c and CD83 antigens for detecting mDCs and mature DCs in a gastric mucosa.

CD11c^+^ mDCs could be recruited from the blood in response to *H. pylori* infection, but we could not observe any association between frequency of circulating mDCs and gastric mucosa mDC density. However, we noted association between frequency of circulating mature CD83-bearing mDCs and gastric mDC density. The accumulation of mDCs in the gastric lamina propria of *H. pylori*-infected patients is necessary for mounting adaptive immune response needed for efficient bacterial clearance. The increased number of mDCs but not CD83-positive DCs in the *H. pylori*-infected gastric lamina propria mucosa could indicate that *H. pylori* infection in children may be associated with local accumulation of immature mDCs (lack of CD83-expressing DCs). The presence of these cells might initiate low protective immune response to *H. pylori.*

We had too small biopsy sections to evaluate other activation marker expressions (HLA-DR and CD80). Instead, we studied the expression of more specific DC maturation markers like LAMP3. We could not detect LAMP3^+^ cells in the gastric lamina propria by immunohistochemistry. However, in six *H. pylori*-infected children, we observed these cells in mucosal epithelium. This marker is absent on immature DCs but rapidly increase upon DCs activation [45], so our results might suggest that some *H. pylori*-infected children have small numbers of activated DCs in gastric epithelium. In *H. pylori*-infected adults, DC-LAMP^+^ cells were also observed but they were located only in the gastric lamina propria [26].

Immature human DCs constitutively express intermediate amounts of CD86 and lack of CD80, CD83, and DC-LAMP [45]. CD80 is exclusively induced on mature DCs while CD86 is already present on immature cells and further upregulated upon stimulation [46]. Terminally differentiated DCs induce specific maturation markers including CD83 and DC-LAMP. Recent studies in adults also showed an increased population of HLA-DR-positive [23], DC-SIGN-positive [26], or DC-LAMP-positive DCs with a semimature phenotype (CD83^+^, CD80^−^CD86^low^) [23, 26] in lymphoid follicles of the gastric lamina propria in the *H. pylori*-infected human gastric mucosa, and these cells were in the same location as follicular FoxP3^+^ Tregs [26]. The presence of CD11c-bearing mDCs but not mature DCs expressing CD83 or LAMP3 molecules in gastric lamina propria in pediatric may promote tolerance to local antigens rather than immunity. The presence of immature DCs and other immature APCs could increase *H. pylori* density in the gastric mucosa. On the other hand, it can also be beneficial, as it may reduce excessive inflammatory activities responsible for gastric ulcers and premalignant gastric lesions in some individuals. The absence of mature DCs in a pediatric gastric mucosa might also explain the lower local proinflammatory responses to *H. pylori* infection in children than in adults [47].

Our study also demonstrated a tendency to increase the density of CD123- (marker of pDCs) positive cells in gastric epithelium as compared to normal controls. This pDC subset may mount both protective and tolerogenic immune responses [48] and can induce Treg differentiation [49]. A previous study in adults showed that gastric CD303-expressing DC (other marker of pDCs) cells were present in a very low number in both *H. pylori*-infected and uninfected individuals however with no significant difference between the groups. Thus, the presence of pDCs in the site of *H. pylori* infection (gastric epithelium) may initiate low protective immune response to *H. pylori* often observed.

## 5. Conclusion

This study shows that although *H. pylori*-infected children had an increased population of mature mDCs bearing CD83 in the peripheral blood, they lack mature CD83^+^ mDCs in the gastric mucosa, which may promote tolerance to local antigens rather than immunity. In addition, this may reduce excessive inflammatory activity as reported for children compared to adults.

## Figures and Tables

**Table 1:** Gastric mucosa histology of *H. pylori*-infected and noninfected children (median score (range) according to the updated Sydney system: 0, none; 1, mild; 2, moderate; 3, marked).

	Antral mucosa				
	MN cells (intensity)	PMN cells (activity)	Atrophy	Intestinal metaplasia	*H. pylori^^^*
Hp+ children	2 (1-3)	1 (0-2)	0 (0-1)	0 (0–0)	1 (1-3)
Hp- children	1 (0-2)	0 (0-1)	0 (0–0)	0 (0–0)	0 (0–0)
*p*	<0.001^∗^	0.003^∗^	0.703	0.751	<0.001^∗^

Abbreviations: MN: mononuclear cells; PMN: polymorphonuclear cells. Hp+ children: *H. pylori*-infected children; Hp- children: noninfected children. ^^^Modified Giemsa staining method. ^∗^Statistically significant differences (Mann-Whitney *U* test).

## Data Availability

The all data used to support the findings of this study are included within the article.
